# Molecular Detection and Epidemiological Features of Selected Bacterial, Viral, and Parasitic Enteropathogens in Stool Specimens from Children with Acute Diarrhea in Thi-Qar Governorate, Iraq

**DOI:** 10.3390/ijerph16091573

**Published:** 2019-05-06

**Authors:** Ali Harb, Sam Abraham, Bertha Rusdi, Tanya Laird, Mark O’Dea, Ihab Habib

**Affiliations:** 1 School of Veterinary Medicine, College of Science, Health, Engineering and Education, Murdoch University, Perth 6150, Australia; 2 Thi-Qar Public Health Division, Ministry of Health, Nassriya 64001, Iraq; 3 High Institute of Public Health, Alexandria University, Alexandria 0203, Egypt

**Keywords:** Iraq, adenovirus, *Salmonella*, *Entamoeba*, *Campylobacter*

## Abstract

Knowledge of etiology causes of diarrheal illness is essential for development and implementation of public health measures to prevent and control this disease syndrome. There are few published studies examining diarrhea in children aged <5 years in Iraq. This study aims to investigate the occurrences and epidemiology of selected bacterial (*Salmonella* spp. and *Campylobacter* spp.), viral (adenovirus, norovirus GI and GII, and astrovirus), and parasitic (*Entamoeba* spp. and *Giardia* spp.) agents in stool samples from 155 child diarrheal cases enrolled between March and August 2017, in a hospital-based cross-sectional study in Thi-Qar, southeastern Iraq. Using molecular techniques and sequence-based characterization, adenovirus was the most frequently detected enteropathogen (53/155 (34.2%)), followed by *Salmonella* spp. (23/155 (14.8%)), *Entamoeba* spp. (21/155 (13.5%)), and *Campylobacter* spp. (17/155 (10.9%)). Mixed infection with *Salmonella* spp. and *Campylobacter* spp. was evident, and the same was revealed between various enteric viruses, particularly adenovirus and norovirus. The most frequent co-infection pattern was between adenovirus and *Campylobacter* spp., in seven cases (7/155 (4.5%)). Whole-genome sequencing-derived typing data for Salmonella isolates (*n* = 23) revealed that sequence type 49 was the most prevalent in this sample set (15/23 (65.2%)). To the best of our knowledge, this study provides the first report on detection and identification of *floR*, *bla_CARB-2_*, and *mph*A antimicrobial resistance genes in *Salmonella* isolated from children in the Middle East region. Logistic regression analysis pointed to few enteropathogen-specific correlations between child age, household water source, and breastfeeding patterns in relation to the outcome of detection of individual enteropathogens. This study presents the first published molecular investigation of multiple enteropathogens among children <5 years of age in Iraq. Our data provide supporting evidence for planning of childhood diarrhea management programs. It is important to build on this study and develop future longitudinal case-control research in order to elaborate the epidemiology of enteropathogens in childhood diarrhea in Iraq.

## 1. Introduction

Diarrheal diseases accounted for 8% of all deaths in children under five years of age in 2016, and this translates to over 1300 young children dying each day, or approximately 480,000 children a year [1]. In Iraq, the impact of war, sanctions, and sectarian violence left a dysfunctional health system and an on-going public health emergency impacting vulnerable sections of the population, particularly children. Several viral, bacterial, and parasitic infections are among the most common causes of acute diarrheal cases in children [2]. Published studies on childhood diarrhea are lacking in Iraq and, therefore, the pathogen spectrum associated with diarrheal disease requires investigation.

Among enteric viruses, rotavirus is the most commonly identified cause of severe diarrhea among children in Iraq, as well as in many developing countries [3,4]. Adenoviruses are also implicated in several viral outbreaks and sporadic cases across all age groups, causing a broad spectrum of clinical symptoms and occurring throughout the year [5,6,7]. Within the adenovirus F subgenera, serotypes HAdV-40 and HAdV-41 are associated with significant outbreaks of disease in infants and children [7]. Other viruses commonly associated with acute gastroenteritis globally include noroviruses (NoVs) and human astroviruses (HAstVs) [8,9]. After enteric viruses, bacterial causes are ranked as the second most common cause of diarrhea in developing countries. *Campylobacter* is a potential etiological agent of bacterial enteritis both in children and adults, and it is second in prevalence to *Salmonella* and similar to *Shigella* in many countries [10]. Non-typhoidal *Salmonella* spp. are among the leading causes of gastroenteritis worldwide, with an increased incidence observed in children less than five years old [11,12]. Invasive cases of non-typhoidal *Salmonella* are frequently reported in infants and young children with a higher risk of secondary complications such as bacteremia and meningitis [13]. In addition, the recent increase of multidrug resistance (MDR) among non-typhoidal *Salmonella* species is a serious problem worldwide, due to the widespread use of traditional antibiotics in human and veterinary medicine, raising global public health concern [14]. Next to viral and bacterial causes, amebiasis and giardiasis are among the major intestinal parasitic infections causing childhood diarrhea in many developing countries [15], and are endemic throughout socio-economically deprived communities [16,17]. Given the multifactorial nature of diarrheal illnesses, it is suggested that enteric pathogen co-infections play an important role in gastroenteritis; however, research efforts often focus on a small range of species belonging to a few pathogen groups [13,14,15,16,17,18]. Thus, studies oriented at investigating the role of co-infections with enteric pathogens in cases of acute diarrhea are required.

In Iraq, the morbidity and mortality associated with diarrhea is high, particularly among children <5 years [4]. Elevated morbidity and mortality is predominantly due to serious challenges facing the delivery of basic public health and environmental sanitation services across Iraq, after decades of war and political instability. Previously, we investigated gastroenteritis caused by *Salmonella* infection among children aged below five years in Thi-Qar, southeastern Iraq [18]. Thi-Qar is one of the least developed and poorest governorates in Iraq, and it is important to investigate the spectrum of infectious causes of children diarrhea in such an unprivileged setting in Iraq. Hence, we transported aliquots of fecal samples from child diarrheal cases recruited in Thi-Qar (Iraq) to the Antimicrobial Resistance and Infectious Disease (AMRID) laboratory at Murdoch University (Australia). The present study is pilot in nature, and aims to conduct a comprehensive molecular screening survey of selected viral, bacterial, and parasitic agents. This molecular-based survey hopes to explore the coexistence between several infectious pathogens, along with their related clinical and epidemiological features among children with acute diarrhea in Thi-Qar.

## 2. Materials and Methods

### 2.1. Study Setting and Design

The study population consisted of children below five years of age presenting with acute diarrhea to the Enteric Diseases Clinic of two referral children hospitals in Thi-Qar, a regional governorate situated in southeastern Iraq, between March and August 2017. This survey is a follow-up from an initial hospital-based cross-sectional study that focused on culture-based screening and characterization of non-typhoidal *Salmonella* [18]. The initial study included 320 diarrhea cases of children below five years; details of case enrolment, stool specimen collection, and questionnaires administered to the child’s parent or guardian to gather information on basic socio-demographic information and potential risk factors for infection are presented in full details elsewhere [18].

For the present study, aliquots of fecal samples from half of the diarrhea cases (*n* = 320) enrolled in the primary study [18] were selected for further molecular screening of a panel of enteropathogens. The decision to select half of the cases was based on feasibility and cost-effectiveness. Random selection of the cases was done using the “select cases” tool in the Statistical Package for the Social Sciences software (SPSS for windows, version 15.0), with the option for selecting approximately 50% of the cases. Thus, in this study, 155 aliquots of stool specimens from children below five years presenting with acute diarrhea were selected for molecular screening of a panel of viral, bacterial, and parasitic enteropathogens.

### 2.2. Stool Samples Processing and DNA Extraction

The sampled stool specimens were kept at 4 °C at the hospital facility, and each sample was divided into two aliquots; one aliquot was placed in Amies transport media with charcoal (COPAN, Italy), labeled and transported under cold chain to the Microbiology Laboratory, University of Thi-Qar, for *Salmonella* detection using the culture-based method [18]. The second aliquot was stored in RNA later® solution (Ambion, USA) as per the manufacturer’s instructions and then shipped from Iraq to Australia. Molecular analysis was conducted at the AMRID Laboratory of Murdoch University. Genomic DNA was extracted from all fecal samples suspended in RNA later® solution using a Bioline Isolate Fecal DNA kit (ISOLATE II, Genomic DNA Kit, Bioline), according to the manufacturer’s recommended protocol. Purified DNA was stored at −20 °C until further analysis. The following panel of enteropathogens was screened (Table 1): (a) *Salmonella* spp. and *Campylobacter* spp. as targeted common bacterial enteric pathogens; (b) adenovirus, norovirus (GI and GII), and astrovirus as representative viral causes of diarrhea; (c) *Entamoeba* spp. and *Giardia* spp. as targeted parasitic causes. In this study, we use the term co-infection to denote cases where different classes of enteropathogens were detected together, for example, bacteria and virus from the same stool specimen. We use the term mixed infection to refer to cases where different agents from the same class were detected together, such as two bacterial species.

### 2.3. Molecular Screening of Enteropathogens

#### 2.3.1. Bacteria

In the present study, the randomly selected aliquots of fecal samples encompassed 23 out of the total 33 non-typhoidal *Salmonella* isolates identified in previous study [18]. We further characterized those 23 non-typhoidal *Salmonella* isolates using whole-genome sequencing (WGS). The WGS was utilized to validate the previous serotype identities of *Salmonella* isolates, to screen and match between antimicrobial resistance genes and the resistance phenotypes, and to screen for multilocus sequence types (MLST). For WGS, library preparation was performed using an Illumina NexTera® XT library preparation kit (Illumina) as per the manufacturer’s instructions. Sequencing was performed on an Illumina Nextseq platform using a mid-output 2 × 150 kit. Reads were de novo assembled using SPAdes 3.11.1 software [19]. Contig files were uploaded to the Center for Genomic Epidemiology (http://www.genomicepidemiology.org/) to screen for MLST and serotypes, and to extract antimicrobial resistance gene data. From all transferred stool aliquots (*n* = 155), we screened for the *S. enterica* gene *invA* using conventional PCR as previously described by Swamy et al. [20]. Screening for *Campylobacter* was undertaken using the conventional PCR assay targeting the 16S ribosomal RNA (rRNA) gene according to protocol described by Barletta et al. [21].

#### 2.3.2. Viruses

Reverse-transcription PCR (RT-PCR) assays were performed using SuperScript III One-Step Platinum@ Taq (Invitrogen, USA) for detection of three enteric viruses: human astrovirus, norovirus group 1 (GI), and norovirus group 2 (GII) [22], while adenovirus was detected by conventional PCR [23]. The specific primers used in this reaction are outlined in Table 1. Bands of the expected size from each assay were excised from 1.5% agarose gels and DNA was purified though filter tips. DNA sequencing was performed at the Australian Genome Research Facility (Perth, WA). The results of the sequencing were analyzed and edited using FinchTV (Version 1.4), then compared to the most similar sequence deposited in public databases on National Center for Biotechnology Information GenBank by applying the Basic Local Alignment Search Tool (BLASTn).

Two randomly chosen samples which were positive on the adenovirus screening PCR were analyzed using WGS, utilizing the same procedures described above. Initial screening for adenovirus genomes was performed using SPAdes, and generation of complete genomes was performed using Geneious V10.2.3 to map raw read data against a representative HAdV-41 genome (GenBank Accession KY316161). Annotation of adenovirus genomes was performed using Geneious V10.2.3. The adenovirus sequences were submitted to NCBI GenBank under the accession numbers MG925782 (MU22) and MG925783 (MU35).

#### 2.3.3. Parasites

A nested PCR assay for the detection of *Entamoeba* species in stool aliquots was utilized according to the procedure previously described by Al-Areeqi et al. [24]. The presence of *Giardia* spp. in all samples was screened at the glutamate dehydrogenase (*gdh*) locus using a quantitative PCR (qPCR) [25].

### 2.4. Statistical Analysis

Descriptive data analysis was used to determine the frequency of enteropathogen occurrence and distribution over a range of variables related to study subjects. Statistical analyses were performed using univariable logistic regression analysis (STATA software package, version 11.0). Univariable logistic regression models were used to examine the correlation between demographic characteristics, household features, and breastfeeding patterns in relation to the binary outcome variable of pathogen detection (presence vs. absence of “a pathogen” of concern in diarrheal stool samples). The analysis examined the correlation between the predictor variables and each of adenovirus, *Salmonella* spp., *Campylobacter* spp., and *Entamoeba* spp. Those four enteropathogens were the most frequently detected in diarrheal stool samples. The analysis did not involve the other enteropathogens detected in less than 10% of the diarrheal samples, nor the various mixed and co-infection combinations detected in low number (minimum = 1, and maximum = 7) of samples.

### 2.5. Ethics and Consent Approval

The study protocol was approved by the Murdoch University Human Research Ethics Committee (Permit No. 2015/224). Permission to conduct the study was also obtained from the Ministry of Health, Iraq (Permit No.11/5/393) and the children’s hospitals in Thi-Qar Governorate (Permit No.1/4/26885). As the study subjects were children under the age of five, informed verbal consent was obtained from their caregivers (parents/guardians) before enrolment. Movement of samples from Iraq to Australia was granted by the Department of Agriculture (Australian Government), under quarantine import permit number 0000369563.

## 3. Results

In this study, we tested a total of 155 stool samples from children with acute diarrhea. Of all cases, the male:female ratio was 1.4:1 and 93 (60%) were under two years of age (Table 2). Descriptive information about demographic characteristics of the cases, their breastfeeding patterns in the first six months of age, and recorded household features, together with information about caregivers’ hygiene practices, is presented in Table 2.

Among all samples, adenovirus was the most frequently detected enteropathogen (53/155 (34.2%)), followed by *Salmonella* spp. (23/155 (14.8%)), *Entamoeba* spp. (21/155 (13.5%),) and *Campylobacter* spp. (17/155 (10.9%)) (Table 3). Those four etiologic agents accounted for 73.4% of the spectrum of enteropathogens detected in the study samples. Group I noroviruses were the least detected (5/155 (3.2%)) among the panel of enteropathogens screened for in this study (Table 3). WGS analysis of two adenovirus PCR positive samples using SPAdes de novo assembly of raw reads returned large contigs consistent with HAdV-41 adenovirus genomes. Raw read files were mapped to the HAdV-41 KY316161 to obtain an entire genomic sequence for each strain. BLASTn analysis of strains MU22 and MU35 demonstrated most homology to existing HAdV-41 strains, with 98.6% pairwise homology to Genbank accessions AB728839 and KY316161, and 98.56% homology to each other.

Results presented in Table 3 highlight the diverse nature of pathogens among cases of acute diarrhea in Iraqi children. Mixed infection with the bacterial pathogens *Salmonella* spp. and *Campylobacter* spp. was evident, and the same was revealed between various enteric viruses, particularly adenoviruses and noroviruses (Table 3). Nevertheless, there was no mixed infection between the two parasitic agents *Entamoeba* spp. and *Giardia* spp. (Table 3). Moreover, co-infection with different classes of enteropathogens was common among the tested samples. Of interest, co-infection with adenovirus and *Campylobacter* spp. was detected in seven cases (7/155 (4.5%)). Co-infection with a bacterial, viral, and a parasitic etiologic agent all together was detected in nine cases (9/155 (5.8%)).

Table 4 summarizes some WGS-derived typing data for *Salmonella* isolates (*n* = 23). A total of four multilocus sequence types (STs) were characterized among the 23 isolates; of which, *S. typhimurium* ST49 was the most common (15/23 (65.2%)). All of the whole-genome sequenced *Salmonella* isolates harbored at least one *tet* gene, with the *tet*B gene having the highest frequency (*n* = 12), followed by *tet*A (*n* = 8) and *tet*G (*n* = 3). Five groups of streptomycin-resistance genes were detected among 17 out of the 23 *Salmonella* isolates, consisting of *aad*A7 (*n* = 5), *str*B (*n* = 4), *str*A (*n* = 3), *aad*A2 (*n* = 3), and *aad*A1 (*n* = 2). For aminoglycoside resistance genes, 11 of the 23 *Salmonella* isolates carried *aph(3’)-Ic* (*n* = 7) and *aac(3)-Id* (*n* = 4). The *sul*1 gene was identified in eight of the sulfonamide-resistant *Salmonella* isolates. Each of *β*-lactamase (*bla*_CARB-2_) and kanamycin (*aph(3’)-Ia*) resistance genes were found in four *Salmonella* isolates. Furthermore, trimethoprim (*dfrA*14), azithromycin (*mphA*), erythromycin (*erm*42), and florfenicol (*floR*) resistance genes were also detected in a few isolates (Table 4).

**Figure 1:**
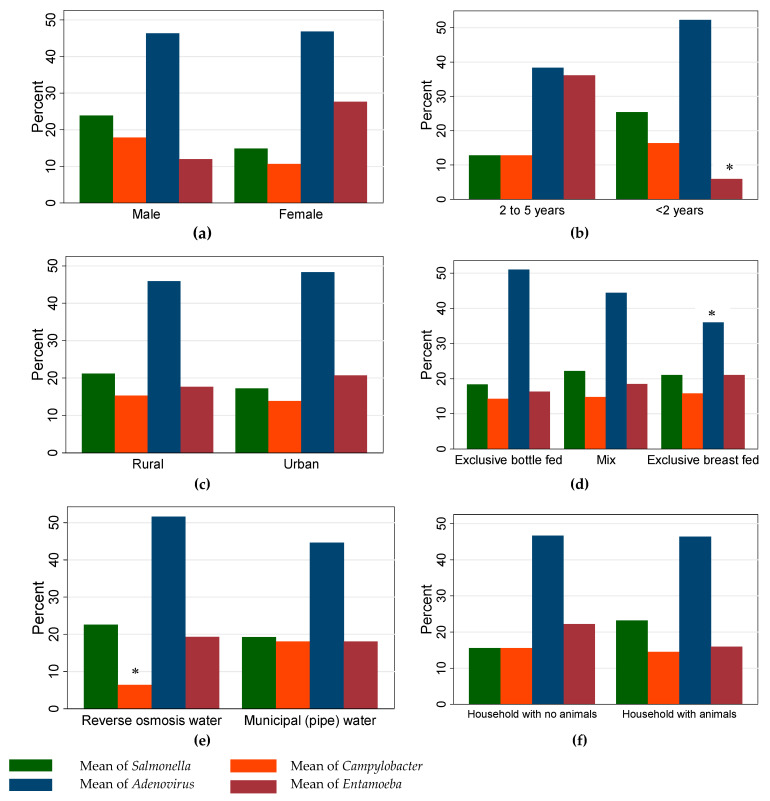
Distribution (percentage of cases) of demographic characteristics (sex (**a**), age (**b**) and residence (**c**)), breastfeeding pattern in the first six months of age (**d**), and household features (water source (**e**) and domestic animals (f)) in relation to frequently (>10%) detected enteropathogens in diarrheal cases (*n* = 155) among children <5 years old. The symbol (*) denote bars of categories with statistical differences.

**Figure 2:**
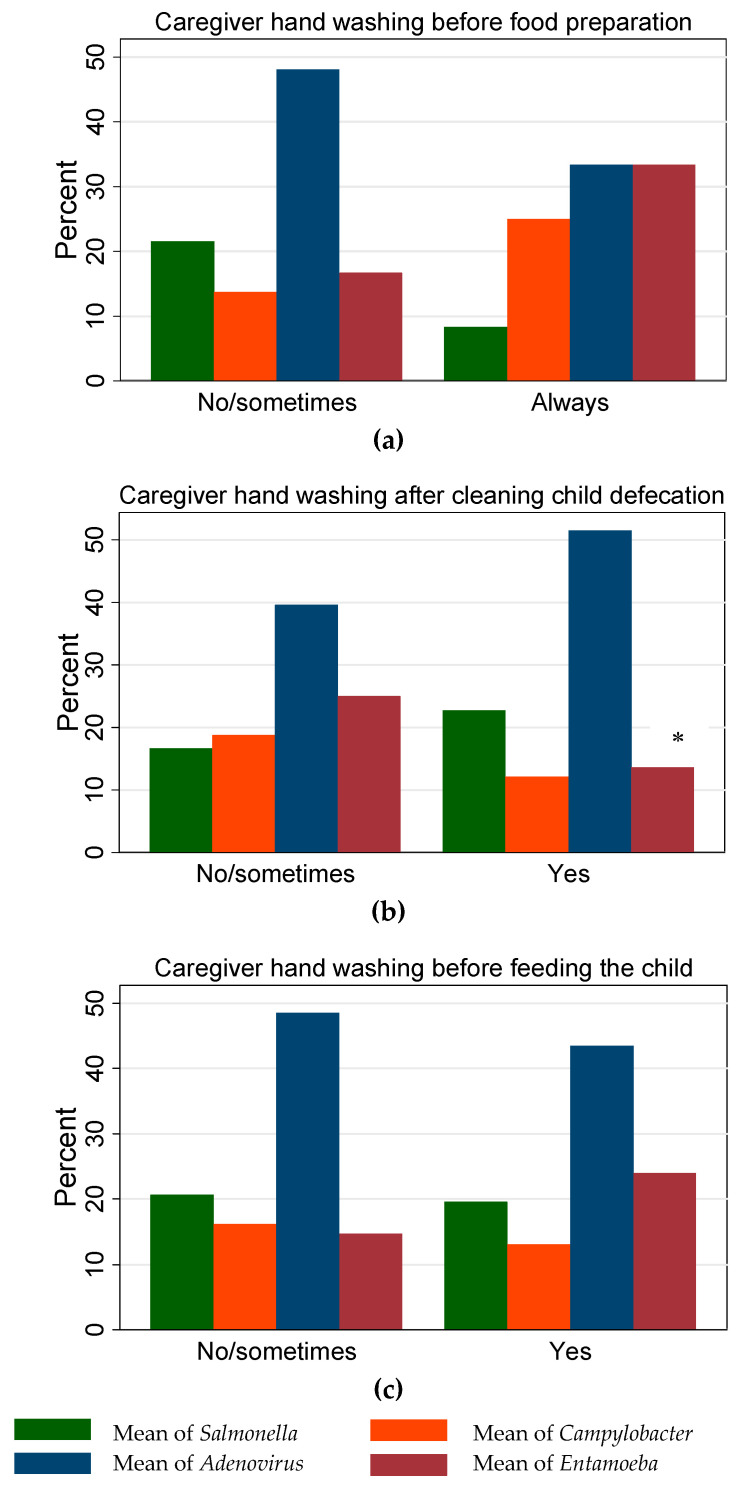
Caregivers’ hygienic practices; (**a**) hand washing before food preparation; (**b**) hand washing after cleaning child defecation; (**c**) hand washing before feeding the child, in relation to percentage of enteropathogens cases (Figure 1) detected in diarrheal children (*n* = 155) <5 years old in Thi-Qar Governorate, Iraq. The symbol (*) denote bars of categories with statistical differences.

Few enteropathogen-specific associations were significant based on logistic regression analysis (Figure 1). Among the cases enrolled in this study, detection of *Entamoeba* spp. was less likely (*p* < 0.001) to occur among children younger than two years (odds ratio (OR) = 0.12, 95% confidence interval (CI) = 0.03–0.37). Lower odds (*p* = 0.051; OR= 0.46, 95% CI = 0.21–0.99) of adenovirus detection were associated with children exclusively breastfed compared to children exclusively bottle-fed. Among the present study subjects, the likelihood of PCR detection of *Campylobacter* spp. in children from households supplied by pipe water was 5.12 (95% CI = 1.12–23.27) times higher (*p* = 0.034) compared to children from households supplied with (purchased) reverse osmosis-treated water. Figure 2 shows the relationship between caregivers’ hygienic practices and occurrence of the four frequently detected enteropathogens in diarrheal cases. According to results from the logistic regression model, the odds of *Entamoeba* spp. detection in children belonging to caregivers who reported always washing hands after cleaning child defecations was three times lower (*p* = 0.030; OR = 0.34, 95% CI = 0.14–0.90) compared to children belonging to caregivers who did not (at all/not always) wash hands.

## 4. Discussion

The majority of enteric bacterial and viral pathogens are not routinely screened for in Iraqi hospitals, due to a lack of basic diagnostics and sufficiently trained personnel [26]. The war in the last decade destroyed substantial capacities of hospitals and public health laboratories in Iraq, and nearly two-thirds of its qualified medical personnel emigrated [27]. In general, published research on diarrheal illnesses in the Iraqi population is very limited, and has mainly focused on screening for single-pathogen infections or, at best, infections with pathogen groups [4,5,6,7,8,9,10,11,12,13,14,15,16,17,18]. In the present study, we report the first molecular epidemiological investigation describing the occurrence and co-existence of several enteropathogens in stool samples from diarrheal children <5 years of age in one of the least developed governorates in Iraq. Added to that, we profiled sequence types and genes conferring resistance to several antimicrobial groups among non-typhoidal *Salmonella* isolated for the diarrheal cases. This study demonstrates the value of WGS as a tool for comprehensive analysis of bacterial and viral pathogens commonly detected in diarrheal patients.

In the present study, human adenovirus (HAdV) was the most common enteropathogen, detected in 53 (34.2%) cases. A survey of patients under five years in Australia from 2007 to 2010 revealed that adenovirus was also the most common cause of gastroenteritis in the studied population, emphasizing the importance of this virus in childhood diarrhea in both developing and developed countries [8]. Detection rates in the present study are considerably higher than published research on HAdV in children with diarrhea from other Middle East and North African countries such as in Kuwait (4%) [28], Qatar (6.25%) [29], Saudi Arabia (8%) [16], and Egypt (20%) [30]. Also, our finding is higher compared to the published occurrence of HAdV in East Asia (9.8–20%) [31] and in Bangladesh (10.7%) [32]. The reasons for the higher HAdV detection rate are not clear. However, it is important to note that the pan-adenovirus PCR assay used in this study detects all adenovirus serotypes and not just the enteric serotypes F40 and F41 [33]. Nevertheless, WGS analysis of two of the PCR positive samples collected in this study allowed the extraction of complete genomes of HAdV-41 serotypes, providing evidence that this enteric serotype, as expected, is circulating in the study population in Iraq. Also worth highlighting is that our study design may have impacted our observed frequency of enteropathogens, including HAdV. Because the diarrheal cases were sampled in the warmer summer months, it is possible our observed frequencies are higher than for other times of year, as described elsewhere [5]. Hence, future case-control studies are needed to accurately predict the frequency of pathogens and potential changes due to seasonality.

Non-typhoidal *Salmonella* was detected in 14.8% of the stool samples from children with diarrhea, and was the second most frequently detected enteric pathogen in this study. This result is consistent with studies conducted previously in Iraq (15%) [34], and in other neighboring countries such as Kuwait (18%) [35] and Saudi Arabia (15.3%) [16]. Our results re-emphasize the importance of non-typhoidal *Salmonella* in the epidemiology of childhood bacterial diarrhea in Iraq. We previously demonstrated that a higher likelihood of positive isolation of non-typhoidal *Salmonella* from children diarrheal cases in Thi-Qar was associated with source of water and presence of domestic animals in the household, as well as with caregiver education level and hygienic practices [18].

*Entamoeba* spp. were found to be the third ranked among the seven enteropathogens screened for in the present research. This finding is consistent with previous surveillance data from Saudi Arabia [16], Oman [36], Yemen [24], and Libya [17], where *Entamoeba* spp. were commonly isolated from children diarrheal samples. Intestinal parasitic infection is a significant public health burden, especially in poor and socio-economically deprived communities [16], which is relevant to the situation in Thi-Qar where 37.8% of the population lives below the poverty line of United States dollar (USD) $2.5 per day [37]. Added to that, the proportion of the population in Thi-Qar using an improved sanitation facility is very low, with only 20.8% utilizing the public sewage system as the primary system, while 39.4% rely on a covered canal outside the house, and 30% primarily use a septic tank [37]. An alarming 54.8% of the population in Thi-Qar disposes of garbage in open areas [37]. A number of case-control and cohort studies on diarrhea in children demonstrated that unsafe water supply and poor sanitation are important risk factors associated with enteric parasite infections [16,17,18,19,20,21,22,23,24].

Very limited research was conducted on *Campylobacter* occurrence in childhood diarrhea in Iraq, as it is not screened for in pediatric hospitals, hampering our understanding of the role of *Campylobacter* spp. in diarrheal illness in this setting. Our results indicate positive PCR detection of *Campylobacter* spp. in 10.9% (17/155) of the screened stool samples from child diarrheal cases. Interestingly, recent findings from the Global Enteric Multicenter Study [38] indicate that the fraction of severe diarrheal cases in infants attributed to *Campylobacter jejuni* or *Campylobacter coli* ranged from 6% in Kenya to 12% in Bangladesh, which is comparable to the present study finding from Iraq. We also conclude, based on logistic regression analysis, that the likelihood of detection of *Campylobacter* spp. in children from households supplied by pipe water was higher compared to those supplied with reverse osmosis-treated water. This finding is in accordance with a cross-sectional study on *Campylobacter* infections among diarrheic children in Ethiopia, where the highest rates of infections were reported in children whose family did not use a protected water source [39]. In spite of growing evidence regarding the burden of *Campylobacter*-attributed diarrhea in developing countries, we know little about what, how, and where children contract infection [40]. Further research is urgently required to investigate the role of supplied household water and the role of domestic animals in the transmission of *Campylobacter jejuni*, especially in populations living with poor sanitary conditions, similar to those in Thi-Qar in south of Iraq.

The spectrum of co-existence of enteric pathogens and their role in diarrheal illnesses could be understood better by utilizing recent advances in diagnostic tools. The utilization of molecular tools in the present study shed light on the potential occurrence of mixed infection between the bacterial pathogens *Salmonella* spp. and *Campylobacter* spp., and the same was revealed between various enteric viruses (Table 3). Children can be exposed to multiple pathogens at home, the playground, and daycare [41]. The presence of mixed infections complicates diagnosis of a specific pathogen responsible for the disease and may result in an additive impact, leading to a more severe clinical disease [42]. Our results also point to an intriguing frequency (4.5%) of co-infection between adenovirus and *Campylobacter* spp. This co-infection pattern should be viewed in parallel with the results of the logistic regression modeling, as our results pointed to higher odds of adenovirus detection in children exclusively bottle-fed (compared to exclusively breastfed), as well as a higher likelihood of PCR detection of *Campylobacter* spp. in children from households supplied by pipe water (compared to reverse osmosis water). In settings where potable water may be limited or surfaces contaminated, cleaning feeding bottles adequately may be impossible, placing infants at a heightened risk of infectious disease. The role water plays in the epidemiology of adenoviruses and *Campylobacter*, as well as the potential health risks constituted by these pathogens in water environments, is widely recognized [43,44,45]. Adenoviruses are considered the only DNA viral pathogens in the enteric virus group. They are robust viruses which are non-enveloped with a double-stranded DNA (dsDNA) genome and are, thus, more resistant in the environment, including water sources, than other enteric viruses [44]. A recent multi-country study suggests that treatment of drinking water and improved sanitation reduced risk associated with *Campylobacter* infection [46]. The frequent co-infection that we report in the present study between adenoviruses and *Campylobacter* warrants a hypothesis that an interaction between hygiene and contaminated water might be a possible route of children co-exposure to both pathogens in Thi-Qar.

Our results demonstrate the usefulness of WGS-derived data in providing in-depth insight into non-typhoidal *Salmonella* isolated for children with diarrhea. To the best of our knowledge, this is the first published WGS-based characterization of *Salmonella* from clinical samples from a Middle-Eastern country. ST49 was the most frequent genotype, followed by ST198 and ST52. The standardization of data and the portable nature of the sequence-based typing allow this method to be used as a worldwide epidemiological tool to study source attribution of enteric pathogens. The three STs characterized in *Salmonella* isolates in our study were recently reported in human salmonellosis cases from neighboring Qatar [47], as well as in the United Kingdom [48]. In several studies, ST49, ST198, and ST52 were also frequently carried in cattle and poultry sources contaminated with *Salmonella*, which might have played an important role in human exposure to infection through food and environmental sources [47,48,49,50].

Analysis of WGS data also revealed that tetracycline, streptomycin, and aminoglycoside resistance genes were commonly harbored by *Salmonella* isolates characterized in this study (Table 4). The emergence and spread of antimicrobial resistance in *Salmonella* is a threat to human public health [14]. The high resistance rates to traditional antibiotics in the current study could be explained by the fact that many of these antibiotics in Iraq, as in other developing countries, are still indiscriminately prescribed in human medicine due to their low cost and wide availability [51]. Three tetracycline resistance genes (*tet*B, *tet*A, and *tet*G) were detected among the sequenced *Salmonella* isolates. A study in Iran also found a similar pattern, as the same three genes were the most commonly identified in tetracycline-resistant *Salmonella* from human stool samples [52]. Florfenicol is a chemosynthesis broad-spectrum antibiotic related to the chloramphenicol class and is mainly used in veterinary medicine [53]. In this study, WGS identified *floR* in 4.3% of *S. enterica* isolates, which is lower compared to findings from a study in Taiwan where *floR* was identified in 19% of the *Salmonella* isolates from children [54]. In the isolates characterized in the present study, *bla*_CARB-2_ and *floR* genes were all associated with *S. typhimurium*, with one exception of a strain of *S. hadar* that harbored the *bla_CARB-2_* resistance gene. A similar finding was demonstrated by Randall et al. [55] who also found *bla_CARB-2_* and *floR* genes to be linked with *S. typhimurium* isolated from humans and animals. Using WGS, Nair et al. [48] observed resistance to azithromycin among *Salmonella* serovars isolated from humans to be linked with the presence of *mph*A gene. This is consistent with our results, which also found two *mph*A genes to be associated with the azithromycin resistance profile. To the best of our knowledge, this is the first report of detection and identification of *floR*, *bla_CARB-2_*, and *mph*A in *Salmonella* isolated from children in the Middle East region.

Effective hand hygiene is essential to prevent the spread of microbes from person to person and reduce cross-contamination from hands to food [56]. In the present study sample, the likelihood of *Entamoeba* spp. detection in children belonging to caregivers who reported always washing hands after cleaning child defecations was significantly lower (compared with those belonging to caregivers who did not wash hands). However, our study data could not conclude a relationship between caregivers’ hygienic practices and occurrence of the other frequently detected bacterial and viral pathogens (Figure 2). It is possible that caregivers’ hygienic practice is a limited route of exposure, compared to other sanitary and environmental routes, and, hence, it did not reveal a tangible relationship among the present study samples. It is worth noting that it is not uncommon to experience difficulty in establishing statistical relationships between hygiene-related factors and infections that are multifactorial in nature, as is often the case for diarrheal illnesses. For instance, recently concluded randomized controlled trials that tested the efficacy of improvements in drinking water, sanitation, and hand washing (WSH) in low- and middle-income countries found no significant effects on gut markers of environmental enteric dysfunction, growth at 18 months of age, or diarrhea incidence in two out of three sites [57]. Lack of statistical significance of individual studies should not be taken as implying that the totality of evidence supports no effect. Hence, it is recommended that mothers should always be encouraged to wash their hands following the use of a toilet, cleaning the child’s bottom after defecation, and before feeding the child, as inadequate hand hygiene can transfer contamination to surfaces and foods in the home [58].

## 5. Conclusions

To the best of our knowledge, this study presents the first published molecular investigation of multiple enteropathogens among children <5 years of age in Iraq. Although this was not a case-control study, the frequency of detection of adenovirus, *Salmonella*, *Campylobacter,* and *Entamoeba* suggests that these organisms are important causes of diarrhea in this population. More information is needed about the sources, modes of transmission and risk factors of enteropathogens in Iraqi children in order to develop methods to control these infections. In future work, it is important to build on the present study and plan for longitudinal case-control research to investigate in depth the epidemiology of enteropathogens in childhood diarrhea, and to perform environmental, water source, and animal sampling. Phenotypic and genotypic characterization of *Salmonella* resistance to some clinically important antimicrobial emphasizes the need to carry out long-term monitoring. Overall, this work fills a gap in research on the frequency of a range of enteropathogens and could be used by public health authorities for informing diarrhea control programs among infants and children in Iraq.

## Figures and Tables

**Table 1:** Primers and probes used for molecular screening of a panel of seven enteropathogens in diarrheal cases (*n* = 155) among children <5 years. F—forward; R—reverse; rRNA—ribosomal RNA.

Target Pathogen	Gene	Sequence (5′ to 3′)	Amplicon Size (bp)	Reference
*Salmonella* spp.	*invA* F *invA* R	TTGTTACGGCTATTTTGACCA CTGACTGCTACCTTGCTGATG	521	[20]
*Campylobacter* spp.	16S rRNA F 16S rRNA R	GGATGACACTTTTCGGAGC CATTGTAGCACGTGTGTC	812	[21]
Astrovirus	PreCAP1 F 82b R	GGACTGCAAAGCAGCTTCGTG GTGAGCCACCAGCCATCCCT	719	[22]
Norovirus GI	G1SK F G1SK R	CTGCCCGAATTYGTAAATGA CCAACCCARCCATTRTACA	330	[22]
Norovirus GII	COG2 F G2SK R	CARGARBCNATGTTYAGRTGGATGAG CCRCCNGCATRHCCRTTRTACAT	387	[22]
Adenovirus	Ad1 F Ad2 R	TTCCCCATGGCICAYAACAC CCCTGGTAKCCRATRTTGTA	482	[23]
*Entamoeba* spp.	E-1 F E-1 R	TAAGATGCACGAGAGCGAAA GTACAAAGGGCAGGGACGTA	439	[24]
*Giardia* spp.	gdf F gdf R Probe	GGGCAAGTCCGACAACGA GCACATCTCCTCCAGGAAGTAGAC TCATGCGCTTCTGCCAG BHQ2	261	[25]

**Table 2:** Descriptive characteristics of diarrheal cases (*n* = 155) among children <5 years.

Variables	Category	No. of Cases (%)
Gender	Male Female	90 (58.1) 65 (41.9)
Age (years)	<2 years 2–5 years	93 (60.0) 62 (40.0)
Mean age (months) ± SD	22.7 (±12.7)	
Residence	Rural Urban	110 (71.0) 45 (29.0)
Breastfeeding pattern in the first 6 months of age	Exclusively bottle-fed Exclusively breastfed Mix—breast- and bottle-fed	60 (38.7) 64 (41.3) 31 (20.0)
Water source in household	Reverse osmosis water Municipal (pipe) water	58 (37.4) 97 (62.6)
Domestic animals in the household	No Yes	62 (40.0) 93 (60.0)
Caregiver hand washing before food preparation	No Sometimes Always	51 (32.9) 86 (55.5) 18 (11.6)
Caregiver hand washing after cleaning child defecation	No Sometimes Always	15 (9.7) 40 (25.8) 100 (64.5)
Caregiver hand washing before feeding the child	No Sometimes Always	36 (23.2) 49 (31.6) 70 (45.2)

**Table 3:** Frequency of isolation, co-infection, and mixed infection patterns of enteropathogens detected in diarrheal cases (*n* = 155) among children <5 years. CI—confidence interval.

Enteropathogens	No. of Cases	% of Cases (95% CI)	Co-Infection (No. of Cases)	Mixed Infection (No. of Cases)
*Salmonella* spp.	23	14.8 (9.6–21.4)	Adenovirus (5) Astrovirus (3) *Giardia* spp. + astrovirus (3) *Giardia* spp. (2) Norovirus GII (1) Norovirus GII + adenovirus (1) Astrovirus + adenovirus (1)	*Campylobacter* spp. (3)
*Campylobacter* spp.	17	10.9 (6.5–16.9)	Adenovirus (7) *Entamoeba* spp. + adenovirus (3) Norovirus GI (2) Norovirus GI + adenovirus (2) Norovirus GII (1)	*Salmonella* spp. (3)
Astrovirus	11	7.1 (3.6–12.3)	*Salmonella* spp.+ *Giardia* spp. (3) *Salmonella* spp. (3) *Giardia* spp. (1)	Adenovirus (2) Adenovirus + norovirus GII (1)
Adenovirus	53	34.2 (26.7–42.2)	*Campylobacter* spp. (7) *Salmonella* spp. (5) *Entamoeba* spp. (3) *Entamoeba* spp. + *Campylobacter* spp. (3) *Salmonella* spp. + *Giardia* spp. (3) *Salmonella* spp. + *Campylobacter* spp. (2)	Norovirus GII (4) Norovirus GI (3) Astrovirus (2) Astrovirus + norovirus GII (1)
Norovirus GI	5	3.2 (1.0–7.3)	*Campylobacter* spp. (2)	Adenovirus (3)
Norovirus GII	10	6.4 (3.1–11.5)	*Salmonella* spp. (1) *Campylobacter* spp. (1)	Adenovirus (4)
*Entamoeba* spp.	21	13.5 (8.5–19.9)	Adenovirus + *Campylobacter* spp. (3) Adenovirus (3) Astrovirus + adenovirus (1)	None
*Giardia* spp.	11	7.1 (3.6–12.3)	Astrovirus + *Salmonella* spp. (3) Adenovirus + *Salmonella* spp. (3) *Salmonella* spp. (2) *Campylobacter* spp. (1) Astrovirus (1)	None

**Table 4:** Whole-genome sequencing (WGS)-derived typing data and resistance phenotype among *Salmonella* (*n* = 23) isolated from diarrheal cases (*n* = 155) among children <5 years.

Serovars	MLST	Resistance Genes	Resistance Phenotypes *^a^*
*S. typhimurium*	ST-49	*tet*B	TET, S
*S. typhimurium*	ST-49	*tet*B	TET, S
*S. typhimurium*	ST-49	*tet*B	TET, ATH
*S. typhimurium*	ST-49	*tet*B	TET, CTX
*S. typhimurium*	ST-49	*tet*B	ATH, NA, CTX
*S. typhimurium*	ST-49	*tet*B	TET, ATH, TS
*S. typhimurium*	ST-49	*tet*B	TET, ATH, CTX
*S. typhimurium*	ST-49	*tet*B	TET, ATH, CTX, CIP
*S. typhimurium*	ST-49	*tet*B	NA, ATH, CTX, TS, S
*S. typhimurium*	ST-3020	*tet*G, *sul*1, *aad*A2, *blaCARB-2*	TET, ATH, CTX, CIP, TS, S
*S. typhimurium*	ST-49	*tet*A, *sul1, aad*A7, *aph(3’)*-Ic, *aac(3)*-Id	TET, ATH, CTX, GM
*S. typhimurium*	ST-49	*tet*B, *str*B, *aph(3’)*-Ic	TET, ATH, NA, S
*S. typhimurium*	ST-49	*tet*B, *str*B, *str*A, *aph(3’)*-Ic	TET, ATH, CTX, CIP, TS
*S. typhimurium*	ST-3020	*tet*G, *aad*A2, *sul1, blaCARB-2, flo*R	TET, ATH, CIP, CRO, TS, NA, GM
*S. typhimurium*	ST-49	*tet*B, *aad*A7, *sul1, aph(3’)*-Ia, *blaCARB-2*	TET, TS, NA, AMP, CRO, ATH, CIP
*S. hadar*	ST-198	*tet*A, *aad*A7, *sul1, aph(3’)*-Ic, *aac(3)*-Id	NA, ATH, CTX, TS, S
*S. hadar*	ST-198	*tet*A, *aad*A7, *sul, aph(3’)*-Ia, *aac(3)*-Id	TET, CRO, CIP, TS, GM
*S. hadar*	ST-198	*tet*G, *aad*A2, *sul, aph(3’)*-Ia, *blaCARB-2, dfr*A14, *erm(42)*	TET, ATH, CIP, NA, GM
*S. hato*	ST-52	*tet*A, *aad*A1, *sul*1, *aph(3’)*-Ic	TET, CRO, S, GM
*S. hato*	ST-52	*tet*A, *str*B, *str*A, *aph(3’)*-Ic, *mph*A	ATH, NA, CTX, TS
*S. hato*	ST-52	*tet*A, *str*B, *str*A, *aph(3’)*-Ic, *mph*A	ATH, CRO, S, NA, TS
*S. muenchen*	ST-1825	*tet*A, *aad*A1, *sul1, dfr*A14	TET, ATH, CIP, S, TS, NA
*S. muenchen*	ST-1825	*tet*A, *aad*A7, *sul1, aph(3’)*-Ia, *aac(3)*-Id	TET, ATH, CIP, S, NA, CTX

*^a^* TET: tetracycline; ATH: azithromycin; S: streptomycin; TS: trimethoprim/sulfamethoxazole; CIP: ciprofloxacin; NA: nalidixic acid; CTX: cefotaxime; CRO: ceftriaxone; AMP: ampicillin; GM: gentamicin.
